# Use of the autism spectrum screening questionnaire for identification of autism spectrum disorders in 8-10 years old georgian children*

**DOI:** 10.1192/j.eurpsy.2021.1688

**Published:** 2021-08-13

**Authors:** M. Zirakashvili, T. Mikiashvili, G. Chvamania, N. Mebonia, M. Gabunia

**Affiliations:** 1 Mental Health, Georgian Academy of Childhood Disability, Tbilisi, Georgia; 2 Medical Sciences, Ilia State University, Tbilisi, Georgia; 3 Psychology, Javakhishvili Tbilisi State University, Tbilisi, Georgia; 4 Psychology, Ilia State University, Tbilisi, Georgia; 5 Department Of Chronic Deaseases, National Center for Disease Control and Public Health, Tbilisi, Georgia; 6 Child And Adolescent Mental Health, Mental Health Center, Tbilisi, Georgia

**Keywords:** ASSQ, Prevalence of autism in Georgia, autism

## Abstract

**Introduction:**

Rising prevalence of autism spectrum disorders highlights importance of research priority of development of effective screening procedures for schoolage children.

**Objectives:**

The study aimed to identify the prevalence of ASD among 8-10 y old schoolchildren in Republic of Georgia.

**Methods:**

In 2019 a cross sectional survey in four main cities of Republic of Georgia was conducted, totally 3rd and 4th grade (8-10 y old) 16654 children from 211 public schools were included. The Autism Spectrum Screening Questionnaire (ASSQ), completed by parents and teachers, was used to determine children at risk for ASD.

**Results:**

16654 (response rate 74%) parents were agreed to participate in the study. Parents and teachers rated 770 (5.0%) and 669 children (4.9%), respectively, as screen positive (in top five percentile). Cut-off scores for 99-95 percentiles (top 1-5%) was defined. Boys were more likely to be rated screenpositive than girls. Share of boys rated in the top 5% by parents is 5.6% compared to 4.3% of girls. Teachers place boys in the top 5% even more frequently – 6.4% versus 3.4% girls. Pairwise correlation coefficients (0.53) revealed moderate correlations between scores and according to p-values (< 0.05) all correlations were statistically significant.

**Conclusions:**

The study defined the cut-off scores of ASSQ for 8-10 y old Georgian children and gender difference in prevalence of risk for ASD. Using the ASSQ was an effective instrument and could be used in school settings to identify children with special needs. *This work was supported by Shota Rustaveli National Science Foundation of Georgia (SRNSFG), grant - FR-18-304.

**Disclosure:**

No significant relationships.

